# Mouse models of the fragile X premutation and fragile X-associated tremor/ataxia syndrome

**DOI:** 10.1186/1866-1955-6-25

**Published:** 2014-07-30

**Authors:** Robert F Berman, Ronald AM Buijsen, Karen Usdin, Elizabeth Pintado, Frank Kooy, Dalyir Pretto, Isaac N Pessah, David L Nelson, Zachary Zalewski, Nicholas Charlet-Bergeurand, Rob Willemsen, Renate K Hukema

**Affiliations:** 1 Department of Neurological Surgery, Room 502C, UC Davis, 1515 Newton Court, Davis, CA 95618, USA; 2 Department Clinical Genetics, Erasmus MC, Rotterdam, Netherlands; 3 NIDDK, National Institutes of Health, Bethesda, MD, USA; 4 University of Seville, School of Medicine, Seville, Spain; 5 Department of Medical Genetics, University of Antwerp, Antwerp, Belgium; 6 UC Davis M.I.N.D. Institute, Sacramento, CA, USA; 7 Department Molecular Biosciences, UC Davis, Davis, CA, USA; 8 Department of Molecular and Human Genetics, Baylor College of Medicine, Houston, TX, USA; 9 Department of Translational Medicine, IGBMC, Illkirch, France

**Keywords:** CGG trinucleotide repeat, *FMR1*, FMRP, Fragile X premutation, FXTAS, Intranuclear inclusions, Mouse models, RNA toxicity

## Abstract

Carriers of the fragile X premutation (FPM) have CGG trinucleotide repeat expansions of between 55 and 200 in the 5′-UTR of *FMR1*, compared to a CGG repeat length of between 5 and 54 for the general population. Carriers were once thought to be without symptoms, but it is now recognized that they can develop a variety of early neurological symptoms as well as being at risk for developing the late onset neurodegenerative disorder fragile X-associated tremor/ataxia syndrome (FXTAS). Several mouse models have contributed to our understanding of FPM and FXTAS, and findings from studies using these models are summarized here. This review also discusses how this information is improving our understanding of the molecular and cellular abnormalities that contribute to neurobehavioral features seen in some FPM carriers and in patients with FXTAS. Mouse models show much of the pathology seen in FPM carriers and in individuals with FXTAS, including the presence of elevated levels of *Fmr1* mRNA, decreased levels of fragile X mental retardation protein, and ubiquitin-positive intranuclear inclusions. Abnormalities in dendritic spine morphology in several brain regions are associated with neurocognitive deficits in spatial and temporal memory processes, impaired motor performance, and altered anxiety. *In vitro* studies have identified altered dendritic and synaptic architecture associated with abnormal Ca^2+^ dynamics and electrical network activity. FPM mice have been particularly useful in understanding the roles of *Fmr1* mRNA, fragile X mental retardation protein, and translation of a potentially toxic polyglycine peptide in pathology. Finally, the potential for using these and emerging mouse models for preclinical development of therapies to improve neurological function in FXTAS is considered.

## Introduction

**Figure 1:**
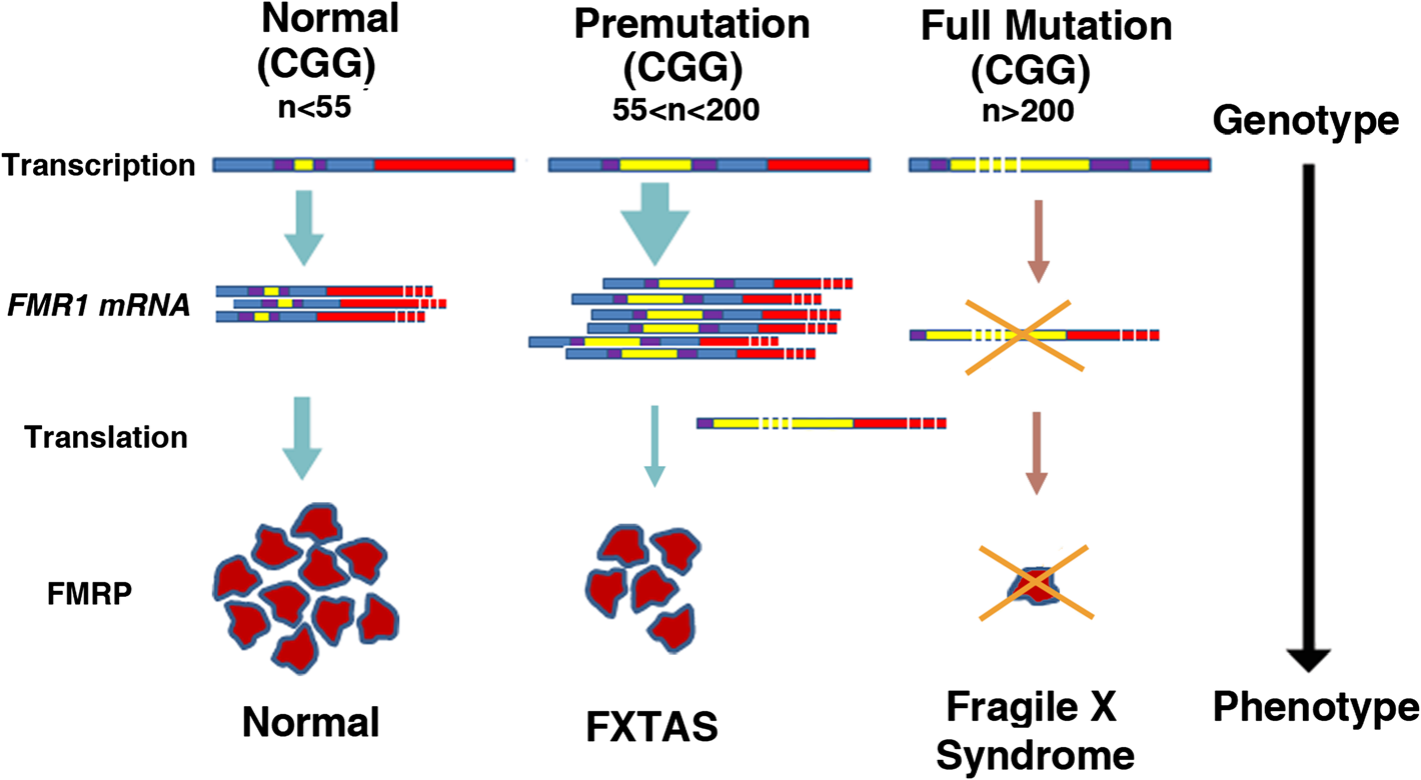
**Most individuals in the general population have between 5 and 54 CGG trinucleotide repeats in the 5′-UTR of *FMR1*.** Repeat length in the fragile X premutation range is 55 to 200, resulting in an elevation in *FMR1* mRNA levels, a moderate decrease in FMRP and an increased risk of developing FXTAS. Repeat size in the full mutation is >200; *FMR1* transcription is silenced due to DNA hypermethylation, and the absence of FMRP results in fragile X syndrome. (Adapted from [9].) FMRP, fragile X mental retardation protein; FXTAS, fragile X-associated tremor/ataxia syndrome.

The Fragile X mental retardation 1 gene (*FMR1*) is located on the long arm of the X-chromosome at Xq27.3 and codes for the fragile X mental retardation protein (FMRP), which is necessary for normal brain development and synaptic plasticity [1-5]. The fragile X gene carries a variable number of CGG repeats in the 5′-UTR of between 5 and 55 in most individuals (modal value 32 to 33; Figure 1). However, due to instability of the repeat across generations, there are large numbers of individuals who carry an expanded CGG repeat of between 55 and 200. These individuals are referred to as fragile X premutation (FPM) carriers, and occur in the general population with an estimated frequency of 1 in 209 females and 1 in 430 males [6,7]. Further expansion of the CGG repeat to greater than 200 in the offspring of FPM carriers leads to the full mutation, silencing of *FMR1* expression and fragile X syndrome (FXS), the major known inherited cause of intellectual disability [4,8].

Carriers with 55 to 200 CGG repeats were originally thought to be clinically unaffected. However, it is now known that they can develop a variety of neurological symptoms, including memory problems, deficits in executive function, depression, anxiety, and problems with numerical processing and magnitude estimates [3,10,11]. They are also at risk for developing the late onset neurodegenerative disorder fragile X-associated tremor/ataxia syndrome (FXTAS). Major symptoms of FXTAS include tremor, ataxia, impairments in executive function and memory, and cognitive decline and dementia in some patients [12,13]. Neuropathology includes brain atrophy, ventricular enlargement, loss of Purkinje neurons, white matter disease, disruption of nuclear lamin A/C architecture and accumulation of intranuclear protein inclusions [5,14].

The chances of developing FXTAS increase dramatically with age, with approximately 45.5% of male and 16.5% of female FPM carriers over the age of 50 developing FXTAS [15]. Indeed, FXTAS may be one of the more common causes of tremor and ataxia in older adults [16]. Besides age, the risk factors that lead to the development of FXTAS in some, but not all, FPM carriers are unknown, but are likely to include CGG repeat length, additional genetic mechanisms and environmental factors (for example, environmental toxins, other illnesses [3]). Identifying the risk factors for FXTAS is particularly important and animal models will undoubtedly play a major role in this area of research.

Because of the increase in the number of people reaching the age of 65, it is likely that the number of cases of FXTAS will increase accordingly, further highlighting the importance of research on FXTAS [16]. Therefore, it is important to understand the underlying pathology in FXTAS, to establish its developmental time course, and to develop rational treatments to delay or halt progression of disease and improve neurological function.

## Review

### Pathogenesis in affected FPM carriers and in FXTAS

**Figure 2:**
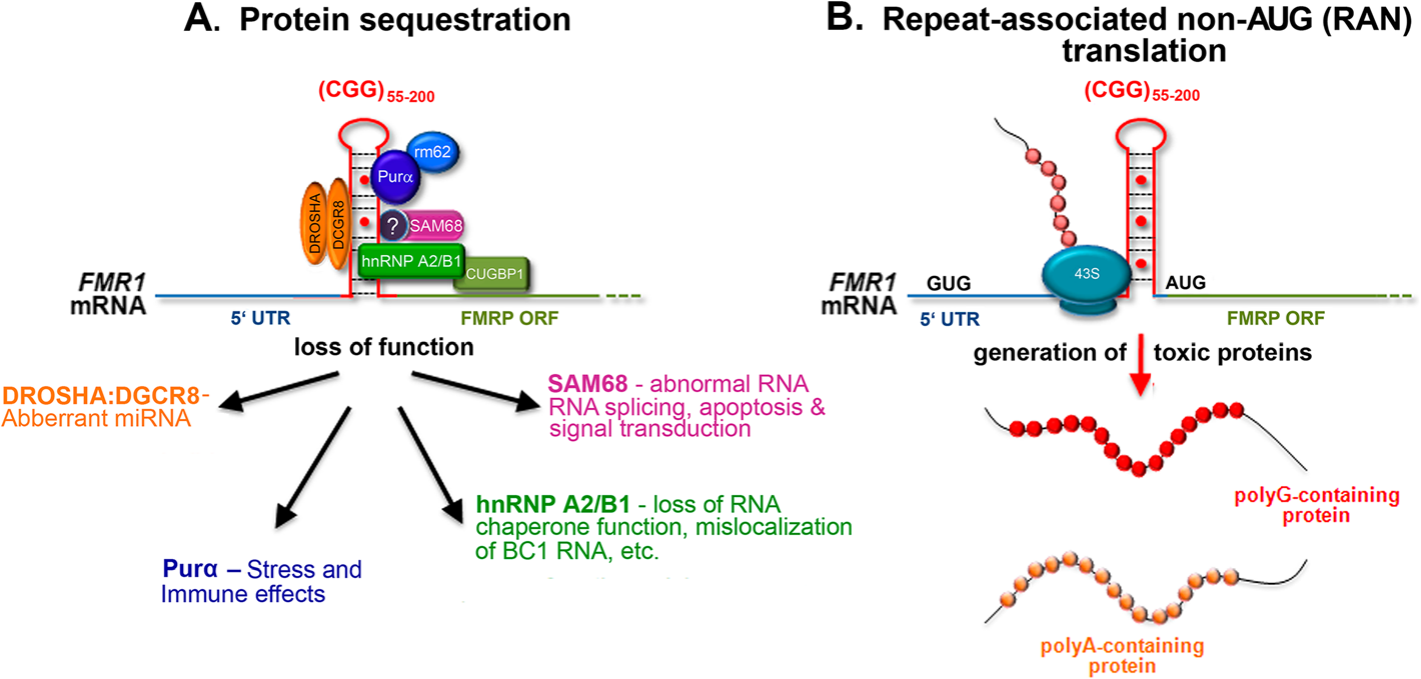
**Potential mechanisms of CGG-repeat RNA toxicity in FMP carriers. (A)** Protein sequestration model: RNA binding proteins are sequestered through their interactions with the expanded CGG-repeat RNA. These proteins can in turn recruit other proteins. The net result of sequestration of these proteins is that they are unavailable to carry out their normal functions and critical cellular processes are thereby altered or blocked. (? -Sequestration of SAM68 by CGG expanded repeats is indirect, presumably through protein-protein interactions). **(B)** Toxic polypeptide model: The 43S translation initiation complex stalls near the CGG repeat hairpin formed on the *FMR1* RNA. This promotes the repeat-associated non-AUG translation of *FMR1* mRNA using a near-AUG start site. This results in a frame shift and the production of polyglycine- and/or polyalanine-containing polypeptides that somehow interfere with normal cell function or may be directly toxic. FMRP, FMRP, fragile X mental retardation protein; ORF, open reading frame; polyA, polyalanine; polyG, polyglycine.

Pathology in affected FPM carriers and in individuals with FXTAS is thought to be the result of RNA toxicity caused by 2- to 8-fold elevated levels of CGG-repeat-bearing *FMR1* mRNA. As depicted in Figure 2A, elevated *Fmr1* mRNA with a CGG repeat expansion is thought to sequester proteins critical for normal cell function, resulting in pathology. This hypothesis is supported by the finding that inclusions isolated from postmortem brain tissue from patients with FXTAS contain *FMR1* mRNA and over 30 proteins, many critical for normal cell function, such as lamin A/C, γH2AX, Sam 68, drosha, Ku86 and hnRNPA2 [17-19]. However, recent findings have suggested an additional model for toxicity, as depicted in Figure 2B, in which a potentially toxic polyglycine-containing peptide is produced as the result of a CGG repeat-mediated non-ATG translation (RAN) mechanism [20]. Research using animal models has provided much of the evidence supporting these theories as presented in this review.

### Development of mouse models

Several mouse models have been developed to study the FPM and FXTAS. These models show much of the pathology associated with CGG repeat expansions on *FMR1*. Table 1 compares pathology seen in FXTAS with that reported in CGG knock-in (KI) mouse models, including molecular, histological and some behavioral deficits. However, no models have been completely successful in reproducing all of the features reported in affected FPM or individuals with FXTAS. An important example is the absence of any reports of obvious tremor in current mouse models, a defining neurological feature of FXTAS. Therefore, it is acknowledged at the outset that current mouse models only partially recapitulate features of the FPM and FXTAS. The mouse models described below have been developed to study specific aspects of disease associated with CGG repeat expansions; each offers advantages and limitations, and each has already provided important insights into disease mechanisms.

**Table 1:** FXTAS compared to the CGG knock-in mouse model

**Pathology**	**Human FXTAS**	**CGG knock-in mouse**
CGG trinucleotide repeat length	55 to 199 CGG repeat length, repeat instability	70 to 300 CGG repeats, modest repeat instability
Elevated FMR1 mRNA expression	Increased 2- to 8-fold	Increased 1.5- to 3-fold
Fragile X mental retardation protein levels	Reduced in several brain regions	Reduced in several brain regions
Motor impairments	Tremor/ataxia, postural sway, parkinsonism	Impaired on rotarod and ladder rung task
Cognitive Impairments	Poor working memory, anxiety, depression, social phobia	Spatial memory deficits, altered anxiety-like behaviors
Intranuclear inclusions	Neurons and astrocytes, highly correlated with CGG repeat length, frequency increases with age	Neurons and astrocytes, related to length of CGG repeat, frequency increases with age

Adapted from [21].

#### ***The Dutch mouse***

**Figure 3:**
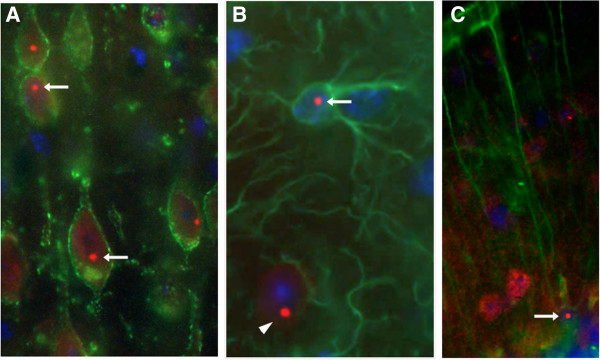
**Ubiquitin-positive intranuclear inclusions in neurons and astrocytes of CGG**_**dut **_**knock-in mice.** White arrows point to red punctate intranuclear inclusions in pyramidal neurons in motor cortex **(A)**, cortical astrocytes **(B)** and Bergmann glia in cerebellum **(C)**. Intranuclear inclusions (red) were labeled by immunofluorescence for ubiquitin, neurons (green) for Kv2.1 potassium channels, and astrocytes and Bergmann glia (green) for GFAP. In **(B)**, note an intranuclear inclusion in an adjacent neuron (arrowhead). Nuclei were stained with DAPI. (Adapted from [25]).

The study of FXS and FXTAS has been greatly facilitated by the development of animal models that mimic much of the pathology associated with these disorders. The first mouse model of FXTAS and the FPM was a CGG KI mouse model from the Willemsen laboratory in the Netherlands, the so-called Dutchmouse (CGG_dut_ KI). This mouse model was generated by replacing the native murine CGG repeat eight trinucleotides in length (CGG8) within the endogenous *Fmr1* gene with a human CGG98 repeat by homologous recombination in embryonic stem cells [22]. Importantly, while minimal changes to the murine *Fmr1* promoter were made when the targeting construct containing the human (CGG)_98_ repeat was generated, the region flanking the repeat in the human *FMR1* was included. These CGG KI mice show moderate instability of repeat length upon paternal and maternal transmission, with both small expansions and contractions (that is, typically fewer than 10 repeats) [22-24]. These CGG_dut_ KI mice have been bred onto a C57BL/6 J background over several generations to establish lines with expanded alleles ranging from 70 to greater than 300 CGG repeats [21,22]. Although expected, based on silencing of *FMR1* expression in FXS, no increased methylation of the *Fmr1* gene has been found even with longer CGG repeat expansions (for example, >300). As described below, these mice models exhibit much of the pathology seen in affected FPM carriers and in FXTAS, including increased expression of *Fmr1* mRNA, decreased FMRP, ubiquitin-positive intranuclear inclusions (Figure 3) and evidence for motor and spatial processing deficits [21].

#### ***The National Institutes of Health mouse***

A second KI mouse was developed at the National Institutes of Health with an initial CGG118 tract [26,27]. The CGG_nih_ KI mice were generated using a different strategy from the CGG_dut_ mice. They were developed using a targeting construct in which exon 1 of the mouse gene was retrofitted with two adjacent but incompatible *Sfi* I sites. The repeats were generated *in vitro* in such a way that they were flanked by the appropriate *Sfi* I sites. This allowed the CGG repeats to be inserted into the mouse locus in the correct orientation and in such a way as to make minimal changes to the mouse flanking sequence. As a result of this strategy, the CGG_nih_ mouse retains the translational TAA stop codon just upstream of the CGG118 repeat that is present in the endogenous murine gene but not the human gene. As with the CGG_dut_ mice, the CGG_nih_ mice show elevated *Fmr1* mRNA levels, decreased FMRP levels, moderate intergenerational expansions, no methylation (even when repeat numbers were >300) and ubiquitin-positive intranuclear inclusions [26].

#### ***Similarities and differences between CGG***_***dut***_ ***and CGG***_***nih***_ ***knock-in models***

The two CGG KI mouse models show similarities as well as some differences [26,28]. Both models show several-fold increases in levels of *Fmr1* mRNA and a reduction in brain levels of FMRP that is inversely related to CGG repeat length. However, they differ in that the reduction in FMRP in the CGG_dut_ KI mouse (20% to 30%) is typically much less than that reported in the CGG_nih_ KI (>50%). Ubiquitin-positive intranuclear inclusions are found in both models, but are more common in neurons and astrocytes in the CGG_dut_ KI model [20]. Inclusions in CGG_dut_ KI mice are widespread in the brain, including the hippocampus, cortex, cerebellum, olfactory bulb, superior and inferior colliculi, and hypothalamus [24]. Purkinje cell loss is seen in postmortem tissue from FXTAS brains, as well as in the CGG_nih_ KI mouse, but has not been reported in the CGG_dut_ KI mouse [26]. Behaviorally, there is evidence for memory impairment in both models [29,30], but the CGG_dut_ KI mouse shows increased anxiety [31] whereas the CGG_nih_ KI mouse shows decreased anxiety [30]. Both models show modest intergenerational repeat instability. Neither model, however, reliably shows large expansions in the length of the CGG repeat tract seen with maternal transmission in FXS, and no methylation or silencing of *Fmr1* expression has been reported in either model. This difference between humans and mice in the frequency of large germline expansions may be due to differences in the length of the perigametic interval in males of both species (that is, weeks), female mice (months) and human females (decades) [32]. The levels of the proteins involved in generating or preventing expansions during the perigametic interval could also contribute to these differences [33].

The reasons for differences between the two models in FMRP reduction, Purkinje cell loss and the frequency of intranuclear inclusions are unclear given that both were generated with CGG repeat sequences that differed only by approximately 20 repeats. However, the cloning strategy used to make these mouse lines differed in that the CGG_nih_ KI mouse retains a greater region of the mouse 5′UTR flanking the CGG repeat, including a TAA stop codon that is not present in the CGG_dut_ KI mouse. The absence of this stop codon in the CGG_dut_ KI may allow RAN translation of a novel polyglycine protein that appears to contribute to CGG repeat toxicity in human cell lines and in a *Drosophila* model [20]; conversely, its presence in the CGG_nih_ KI may block this CCG RAN translation. The ability to compare the pathology between the two mouse models represents an important and powerful tool for understanding the mechanisms of disease in the FPM and in FXTAS.

#### ***Ectopic expression of an expanded CGG90 in transgenic mice***

**Figure 4:**
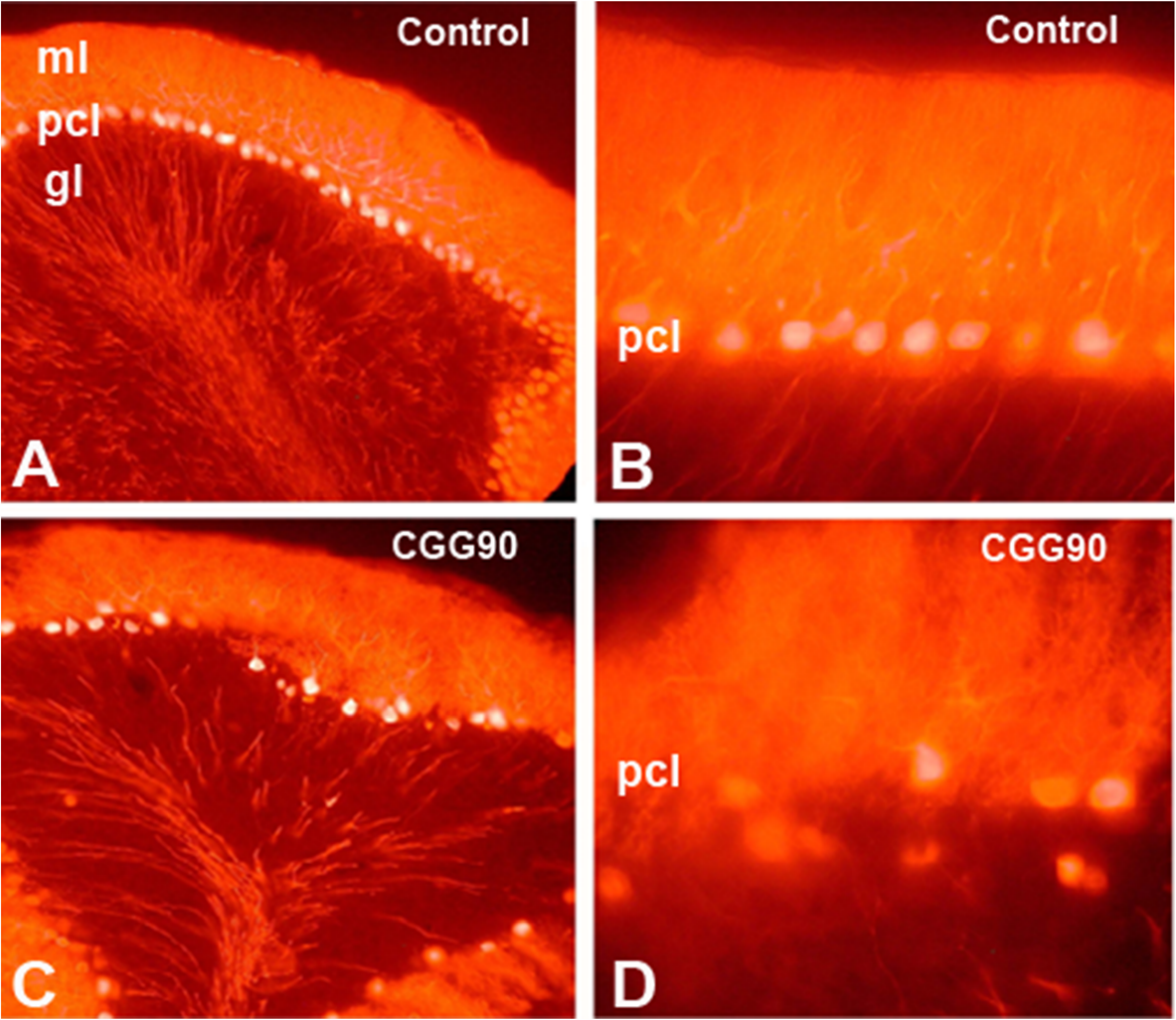
**Ectopic expression of a CGG90 repeat results in Purkinje cell loss. (A)** Cerebellum of control mouse without a CGG90 repeat (that is, L7*Fmr1*) showing normal distribution of Purkinje cells in the Purkinje cell layer. **(B)** Higher magnification of the Purkinje cell layer in control mouse. **(C)** Selective Purkinje cell loss in 32-week-old mouse expressing a CGG90 repeat under the L7 Purkinje cell-specific promoter (that is, L7CGG90*Fmr1*). **(D)** Purkinje cell loss in shown at higher magnification in L7CGG90*Fmr1* mouse. gl, granule cell layer; ml, molecular layer; pcl, Purkinje cell layer. (Adapted from [34]).

In order to determine whether ectopic expression of an expanded CGG90 repeat causes neurodegeneration in the cerebellum, transgenic mice (L7-CGG90-*Fmr1*) were developed in which expression was spatially restricted to cerebellar Purkinje neurons using the L7 promoter [34]. In these mice, the CGG90 repeat was upstream of either *Fmr1* or enhanced green fluorescent protein (EGFP) cDNA (L7-CGG90-*Fmr1*, L7-EGG90-EGFP), with control mice expressing *Fmr1* or EGFP but without a CGG90 repeat expansion (L7-*Fmr1*, L7-EGFP). Significant Purkinje cell loss was observed in 32-week-old L7-CGG90-*Fmr1* and L7-CGG90-EGFP mice compared to wild-type (WT) littermates or L7-*Fmr1*/L7-EGFP mice (Figure 4). Ubiquitin-positive intranuclear inclusions were found in Purkinje neurons of both the L7-CGG90-*Fmr1* and L7-CGG90-EGFP lines, but were not found in either WT littermates or the L7-*Fmr1* or L7-EGFP control lines. Lack of inclusions in control mice, in addition to their presence in the L7-CGG90-EGFP line, demonstrates an essential role for the CGG repeat expansion in inclusion formation, and that expressed CGG repeat containing RNA is sufficient to induce inclusions. These Purkinje neurons stained positive for the 20S core complex of the proteasome, Hsp40, and Rad23B. Interestingly, staining was negative for Purα, hnRNPA2/B1, Tau and α-synuclein - all proteins that have been reported in human intranuclear inclusions in human FXTAS [18]. Motor performance on the rotarod was also impaired in mice expressing the CGG90 repeat compared to controls, and this impairment was not age-related, as similar impairment was seen in 20- and 40-week-old mice. These results provide evidence that CGG repeat mRNA expression is sufficient to cause Purkinje neuron dysfunction and loss similar to that reported in FXTAS [35].

Neuropathological observations to date demonstrate a connection between formation of intranuclear inclusions and cell death. While it is tempting to speculate that the formation of inclusions is the cause of cell loss, such a conclusion is contingent upon understanding what the functional ramifications are when proteins and their interacting partners are sequestered within an inclusion body. A *Drosophila* model ectopically expressing premutation-length CGG repeats showed a neurodegenerative eye phenotype and *Hsp70*/*ubiquitin*-positive inclusions [36]. A subsequent genetic screen showed that CELF1 (CUGBP1), when ectopically expressed, was able to suppress the neurodegenerative eye phenotype [37]. CELF1 was also shown to directly interact with hnRNPA2/B1, known to be present in inclusions of patients with FXTAS [18]. CELF1 is up-regulated overall in the presence of CUG repeats >50, contributing to the mis-regulation of mRNA splicing and translation and the muscle atrophy and weakness observed in muscular dystrophy type 1, the disease for which its involvement is best known [38-40]. CELF1 is therefore predicted to be one potential modifier of CGG repeat-mediated neurodegeneration. Preliminary findings in mice show modulation of neuropathological phenotypes previously reported in the L7CGG90 transgenic mice when the expression of CELF1 is altered (Zalewski *et al*. Abstracts of the 1st Premutation Meeting, Perugia, Italy, 2013). Such findings support an RNA toxicity mechanism (see Evidence for current disease models section), specifically that the sequestration of such proteins within an inclusion inhibits their normal function, leading to dysregulation (at least at the level of RNA processing) in the cell and, over time, cell death.

#### ***Fmr1 overexpressing mice***

Levels of *FMR1* mRNA bearing an expanded CGG are elevated several fold in premutation carriers and in patients with FXTAS, supporting the hypothesis that pathology is the result of *FMR1* mRNA toxicity. However, the possibility exists that toxicity could be due to either the CGG repeat itself, elevated *FMR1* mRNA independent of the repeat expansion, or both. In a *Drosophila* model of FXTAS, high expression levels of a CGG60 repeat causes formation of ubiquitin-positive inclusions and neurodegeneration in the retina in a dosage- and repeat length-dependent manner, whereas moderate expression of the repeat allele results in little pathology. These findings support the notion that overall abundance of a CGG repeat molecule may be important for generating a pathological phenotype [36]. To investigate the potential deleterious effects produced by overexpression of *FMR1* mRNA with a normal CGG repeat length, transgenic mice that overexpress *FMR1* mRNA bearing a normal length CGG29 repeat have been generated [41]. The CGG29 transgenic mouse was obtained by pronuclear injection of a construct containing the human *FMR1* cDNA with 29 CGG repeats under control of a SV40/T7 promoter. This model results in a 20- to 100-fold increase in *FMR1* mRNA in all tissues studied (for example, liver, cerebral cortex and cerebellum). However, these animals did not show significant differences from WT mice in general activity or anxiety-related behaviors in open-field tests. These results suggest it is expression of the expanded CGG repeat that is primarily responsible for pathology, and not overexpression of *Fmr1* mRNA *per se*. Other transgenic mice overexpressing *FMR1* mRNA have been made using a yeast artificial chromosome (YAC) containing the full-length human *FMR1* gene. These YAC mice show a 2- to 3-fold increase in expression of *FMR1* mRNA and a 10- to 15-fold increase in FMRP compared with control littermates [42,43]. When crossed with a knock-out (KO) mouse model of FXS that lacks FMRP, some of the pathological features of FXS were reversed. Importantly there were no changes in overall brain morphology at the light microscopic level due to overexpression of mRNA or protein. However, overexpression in otherwise WT mice (that is, not KO mice) also resulted in some abnormal behaviors, including decreased activity, increased anxiety-like behavior and enhanced startle response. Although the authors attributed these behavioral effects to overexpression of FMRP, the high levels of *Fmr1* mRNA could also have contributed to the behavioral effects [43].

#### ***Yeast artificial chromosome transgenic mouse models of the FPM***

YAC transgenic mouse lines have also been generated to study CGG repeat instability [44]. These mice were generated using a CGG92 allele isolated from an adult male premutation carrier, a CGG repeat length that would be expected to show expansion to the full mutation when transmitted through the female germ line in humans. The CGG92 region, including several hundred base pairs of flanking sequence, was cloned into a YAC and purified YAC DNA was injected into FVB/N mouse oocytes and then transplanted into foster mothers. A line of offspring (line TG296) carrying a CGG90 repeat were then identified. Although not yet well characterized, these YAC mice show modest intergenerational CGG repeat instability, expansion and contraction of one to three trinucleotides across generations. There was no influence of parental sex or age on transmission of the repeat.

#### ***New mouse models***

Continued development of new mouse models to study the FPM and FXTAS has resulted in generation of a doxycycline-inducible mouse line with a CGG99 repeat RNA under control of a doxycycline-responsive promoter (R. Hukema, Abstracts of the 1st Premutation Meeting, Perugia, Italy, 2013). Preliminary findings in this mouse show the presence of doxycycline-inducible ubiquitin-positive intranuclear inclusions in the hippocampus and cerebellum. This mouse is being used to determine critical periods for the onset of pathology as well as to help define molecular targets for development of future treatments.

### Brain and cellular pathology

The description of brain pathology associated with FPM and FXTAS is limited by the availability of tissue for analysis. As a result, virtually all that is known about such pathology has come from studies of postmortem tissue from premutation carriers who developed FXTAS, and from findings in animal models. To date there have not been any published studies on brain pathology seen in FPM carriers without FXTAS, including if and when intranuclear inclusions and cell loss (for example, Purkinje neurons) may be occurring.

#### ***Intranuclear inclusions***

The hallmark histopathology in FXTAS includes the presence of ubiquitin-positive inclusions in neurons and astrocytes that is widespread throughout the brain. As a further parallel between human FXTAS and the CGG KI mice, both show the presence of ubiquitin-positive intranuclear inclusions in many regions of the brain [24-26,45]. The CGG_dut_ KI develops intranuclear inclusions in neurons in the cerebral cortex, olfactory nucleus, parafascicular thalamic nucleus, medial mammillary nucleus and colliculus inferior, cerebellum, amygdala and pontine nucleus cortex, hippocampus, hypothalamus, and in granule cells of the cerebellum (Figure 3) [24,28]. Inclusions in the dentate gyrus of the hippocampus are evident as early as 12 weeks of age [29]. The number of inclusions in glia, including astrocytes and Bergmann glia, and their distribution in the brain are more limited, and not as numerous as found in postmortem FXTAS brain tissue [14,25]. In addition, the size of the inclusions correlates significantly with the age of CGG_dut_ KI mice, with smaller inclusions found in younger mice. Interestingly, the gradual increase in the size of the inclusions and the percentage of ubiquitin-positive neurons appear to parallel the progressive development of the neurological phenotype of FXTAS in humans [16]. Brain regions showing the presence of intranuclear inclusions correlate with the clinical features in patients with symptomatic FXTAS. Importantly, inclusions are not limited to the nervous system, and are found in both human FXTAS and in the CGG_dut_ KI mouse in a variety of other tissues, including pancreatic, thyroid, adrenal gland, gastrointestinal, pituitary gland, pineal gland, heart and mitral valve. Inclusions were also found in the testes, epididymis and kidney of patients with FXTAS, but not in the KI mice [46]. Therefore, FXTAS should be considered a multi-organ disease. Systematic analysis of these inclusions shows the presence of more than 20 proteins including ubiquitin, molecular chaperone Hsp40, 20S proteasome complex, DNA repair-ubiquitin-associated HR23B factor and SAM-68, DGCR8, and DROSHA [18,19,24,47-49]. The inclusions also contain *FMR1* mRNA, but surprisingly not FMRP [18]. Similar studies on the protein composition of inclusions found in CGG mouse models have not been carried out, but it is already apparent that there are some similarities between the inclusions in FXTAS and mouse models, including the presence of ubiquitin, SAM68, DGCR8 and lamin A/C, as well as several differences [18,19,24,27,47,50]. Purα has been detected in intranuclear inclusions in a *Drosophila* model of the premutation and overexpression can suppress CGG repeat-mediated neurodegeneration. However, purα has not yet been detected in inclusions in murine models and evidence for its presence in human inclusions is inconclusive [18,50]. Similarly, hnRNP-A2/B1 are found in the intranuclear inclusions in FXTAS [18], but little or none has been found in CGG KI mice [34]. Additional research on the composition of intranuclear inclusions in FXTAS and mouse models would clearly be of value.

#### ***Cell loss***

An important neuropathological finding in human FXTAS is the presence of Purkinje cell degeneration [35]. This has also been observed in the CGG_nih_ KI mouse, and in mice with an ectopic CGG90 repeat expansion whose expression is limited to cerebellar Purkinje neurons as shown in Figure 4[26,34]. However, the generalized brain atrophy, including enlarged ventricles, that has been reported in some patients with FXTAS has not been systematically examined in any of the existing mouse models. Such studies need to be carried out using structural magnetic resonance imaging and quantitative stereology of neurons in brain regions known to be affected in FXTAS, to establish whether similar pathology also occurs in mouse models.

#### ***White matter disease***

FXTAS is also characterized by white matter disease, including loss of glial cells, enlarged astrocytes, spongiosis and pallor in subcortical and cerebellar white matter, including in the middle cerebellar peduncle [14,35,51]. Additional pathology in FXTAS is seen on T2-weighted magnetic resonance images that show hyperintensities in white matter tracts, including the middle cerebellar peduncle [52]. Tractography studies using diffusion-weighted magnetic resonance imaging have provided additional evidence for degeneration in major white matter fibers tracts in FXTAS, including the middle cerebellar peduncle, superior cerebellar peduncle and corpus callosum, that was not found in premutation carriers without FXTAS [51]. As yet, these important findings have not been systematically examined in mouse models of the FPM or FXTAS, and there are no published reports of white matter pathology or degeneration of major fiber tracts in animal models.

#### ***Dendrite and dendritic spine morphology***

Studies of Golgi-stained neurons have also revealed ultrastructural changes in dendrites and dendritic spines in both CGG_dut_ and CGG_nih_ KI mice [30,53]. The CGG_dut_ KI mouse shows fewer dendritic branches proximal to the soma, reduced total dendritic length and longer dendritic spines on basilar, but not on apical dendrites in pyramidal neurons in the primary visual cortex. Neither total dendritic spine density, nor the density for specific dendritic spine subtypes (that is, stubby, mushroom, filipodial) differed between WT and KI mice. Dendrite and dendritic spine morphology has also been examined in CGG_nih_ KI mice in several brain regions, including the medial prefrontal cortex, hippocampus and basal lateral amygdala. In all three brain regions, the branching complexity of apical and basilar dendrites was significantly lower and spines were longer in KI mice compared to WT, consistent with findings in the CGG_dut_ KI mouse. However, in the CGG_nih_ KI mouse, dendritic spine density was generally increased in all three brain regions in contrast to the CGG_dut_ KI mouse, which did not show changes in spine density. It is interesting to note that longer dendritic spines found in the cortex of CGG KI mice have also been reported in Golgi studies of postmortem tissue in FXS [54,55] and in *Fmr1* KO mice [56,57], whereas the reduction in dendritic branching complexity in CGG KI mice was not found in the *Fmr1* KO mouse [56]. The reasons for these similarities and differences are unknown but should be further investigated. To our knowledge, dendritic branching and spine morphology have not been examined in postmortem tissues from carriers of the FPM or patients with FXTAS.

#### ***Lamin A/C disruption***

Expression of expanded CGG RNA also results in the widespread disruption of lamin A/C proteins with associated abnormalities in nuclear envelope morphology *in vitro* and *in vivo*[58,59]. Lamins A/C are intermediate filament proteins that that line the inner nuclear membrane where they help maintain the shape and mechanical integrity of the nucleus [60]. They are generated from a single *LMNA* gene by alternative splicing, and mutations have been linked to a variety of neurodegenerative diseases. Cells deficient in lamin A/C show decreased survival and defective response to DNA damage [61].

These observations suggest that FXTAS may result in a functional laminopathy. This is consistent with recent findings that demonstrate that laminopathy diseases, including restrictive dermopathy and Hutchinson-Gilford progeria syndrome, result in increased levels of reactive oxygen species and accumulation of DNA damage [62]. Moreover, several proteins involved in telomere maintenance [63-65] are present in the intranuclear inclusions characteristic of FXTAS (for example, lamin A/C, Ku80, γH2AX) [18] and could account for shorter telomere length demonstrated in patients with FXTAS [66,67]. Shorter telomere length could also contribute to the reduce life expectancy associated with longer CGG repeat lengths in patients with FXTAS [14,25]. While disruption of nuclear lamin A/C architecture has been reported in mouse embryonic fibroblasts from CGG_dut_ KI mice, studies in mice examining Ku80 and γH2AX have not been carried out [58].

#### ***Mitochondrial dysfunction***

Several symptoms reported in FXTAS share some commonalities with mitochondrial respiratory chain enzyme deficiencies, including gait ataxia, white matter disease, peripheral neuropathology, muscular weakness and neuropsychiatric disorders [68]. Mitochondrial dysfunction occurs in FPM and FXTAS and has been examined in cultured skin fibroblasts and in frozen frontal cortex from postmortem brain tissue samples from premutation carriers with or without FXTAS [68]. Decreased NAD- and FAD-linked oxygen uptake rates have been found in premutation carriers compared to controls. In addition there is reduced expression of the mitochondrial protein MnSOD, an antioxidant enzyme, and nitration of ATPB, a putative marker for nitrative/oxidative stress is elevated approximately 2-fold in FPM and FXTAS compared to controls, indicating mitochondrial dysfunction. Mitochondrial dysfunction has also been found in cultured hippocampal neurons isolated from CGG_dut_ KI mice as early as 4 days *in vitro* (DIV) [69]. Density and mobility were assessed by time-lapse imaging of mitochondria labeled with Mitotracker Red CMXRos, and oxygen consumption was estimated by measuring the rate of change of dissolved O_2_ in the medium surrounding the cultured hippocampal neurons using a Seahorse Bioscience extracellular flux analyzer. CGG_dut_ KI mice showed reduced density of mitochondria in proximal neurites (that is, within 25 μm of soma), as well as significantly reduced mobility compared to WT mice. Neurons from CGG_dut_ KI mice also showed high basal oxygen consumption rates and evidence for increased protein leakage and higher ATP production. The authors suggested that these abnormalities in mitochondrial distribution and bioenergetics may contribute to previous reports of lower viability and reduced dendritic branching of cultured hippocampal neurons [70] as well as to reduced dendritic branching and altered spine morphology in CGG KI mouse neocortex [30,53]. It is important to consider the possibility that mitochondrial disease may contribute to the risk for premutation carriers to become symptomatic or to develop FXTAS, and this potential link should be explored in futures studies in using mouse models.

### Molecular findings

#### ***Fmr1 mRNA and FMRP***

Both the CGG_dut_ KI and the CGG_nih_ KI mice have proven to be very useful models to study the molecular aspects of the expanded CGG repeat. The brains of these two mouse lines show small (10% to 30%) to moderate (>50%) reductions in FMRP, respectively, despite the fact that 2- to 3-fold elevated levels of *Fmr1* mRNA are found [23,26,28,71-73]. These results parallel to a great extent what is found in some human premutation carriers and in patients with FXTAS as outlined in Table 1[21]. The linear correlation between *FMR1* mRNA levels and the repeat size in FPM and in patients with FXTAS [72,74] has also been found in brain tissue from the CGG_dut_ KI mouse [73]. Entezam *et al*. were able to show a direct relationship between CGG-CCG repeat size and *Fmr1* mRNA levels in the brains of the CGG_nih_ KI mice, although the number of mice studied for the different repeat sizes was limited [26]. The cellular mechanism underlying the increase in *Fmr1* mRNA levels is unknown, but could be due to a feedback mechanism resulting from reduced levels of FMRP. Mechanisms underlying reduced FMRP include impeded migration of the 40S ribosomal complex along the expanded CGG tract, as well as the use of an alternative internal ribosome entry site for initiation of translation. An internal ribosome entry site has been identified in the 5′UTR of *FMR1* mRNA [75].

#### ***Fmr1 splice variants and FMRP isoforms***

The *FMR1* gene has 17 exons with alternative splice sites on exons 12, 14, 15 and 17 that result in the expression of multiple FMRP isoforms [76-78]. The splicing pattern of these isoforms is of interest as, in some isoforms, the truncation or absence of functional domains would suggest a change in FMRP functional properties including its selection of protein partners and mRNA targets and its cellular localization. For instance, the N-terminus of FMRP harbors a nuclear localization signal and *FMR1* mRNA binding activity is driven by two K Homology domains encoded by exons 8 to 12 and an RGG box domain in exons 14 to 15 [79]. Additionally, a nuclear export signal is localized to exon 14 and serine phosphorylation sites involved in translational regulatory activity of FMRP as well as methylation sites are also localized to exon 15. The transcript levels of these isoforms are developmentally regulated in the brain of the WT C57BL/6 mouse strain [77], the same strain used to construct the CGG_dut_ KI mouse model [24]. Isoform distributions were similar across 11 different brain regions with the exception of the hippocampus and the olfactory bulb. Although to date no information is available on isoform distribution in the CGG_dut_ KI mouse, the polyadenylation state of *Fmr1* transcripts, which can be informative for the stability and the translational efficiency of the mRNA, has been investigated in these mice. The CGG_dut_ KI mouse exhibits an increased population of short poly(A) mRNAs, usually indicative of inefficiently translated transcripts, compared to WT [80]. It would be interesting to know whether particular mRNA isoforms are thus more efficiently translated than others in the CGG_dut_ KI background.

#### ***Expression profiling***

Dysfunction of the GABAergic system has been reported in CGG_dut_ KI mice [81]. Specifically, overexpression of genes for several GABA_A_ receptor subunits (for example, α1,3,4; β2; γ2) and proteins involved in GABA metabolism (gad1, ssadh) has been observed in the cerebellum, but not the cortex, of CGG_dut_ KI mice, which could be related to the motor phenotype observed in FXTAS [82,83]. In *Fmr1* KO mice, expression was decreased for some of these same genes (for example, *gad1*, *ssadh*), but the reasons for this difference are unclear. Microarray analysis in the cerebellum of transgenic mice that overexpress human *FMR1* with a normal range CGG29 repeat has also been carried out, but there were no clear changes in the GABAergic system compared to controls. Among GABA-related genes, only up-regulation of the GABA_A_ receptor-associated protein-like 2 (*Gabarapl2*) gene was observed [41]. These results provide additional support that pathology in CGG KI mice, at least in the GABA system, is due to expansion of CGG repeats rather than increased mRNA levels, given that *FMR1* mRNA levels were increased 20 to 100 times in these transgenic mice compared with those of WT littermates. However, other changes were seen in the transcriptome of these mice that could be a consequence of an overabundance of *FMR1* mRNA. Interestingly, the two most altered genes in the transcriptome were *transthyretin* (*Trt*), and *serpina3*, putative biomarkers for Alzheimer’s disease [84,85]. Serpina3, a serine protease inhibitor that is released during inflammatory responses, was up-regulated and may reflect the increased prevalence of autoimmune disease (for example, lupus, multiple sclerosis, fibromyalgia, thyroid disease) in females with the *FMR1* premutation [86]. Transthyretin, a transport protein for retinol and thyroxine thought to contribute to thyroid hormone homeostasis, was down-regulated [87]. Although speculative, reduced transcription could be related to the hypothyroidism reported in some patients with FXTAS [3]. In addition, two microRNAs, mir-181a-1 and let-7 appeared up-regulated in CGG mice. Up-regulation of Let-7 miRNA has been also been reported in a *Drosophila* model of FXTAS [88]. This is important because several miRNAs are up-regulated in human premutation carriers [89], although they differed from those observed in CGG transgenic mice [41].

### Electrophysiological findings

#### ***GABA/glutamate imbalance and abnormal synaptic network activity***

The origin of pathology in FXS and in some FPM carriers, with or without FXTAS mutations, is the presence of a CGG repeat expansion on *FMR1*, raising the possibility that some of the same molecular pathways could be affected in both disorders, and those associated with glutamatergic signaling in particular [1,74,90,91]. This is in spite of differences in the causal molecular underpinnings in the disorders, and specifically the lack of FMRP expression in FXS versus the overexpression of *FMR1* mRNA in the FPM and FXTAS. In fact, the dysregulation in excitatory and inhibitory neurotransmission in the central nervous system of FXS KO mice has been the subject of active investigation during the last decade, and evidence has recently surfaced that suggests a similar dysregulation in the CGG KI mice [1,90,91].

Hippocampal CGG_dut_ KI neurons *in vitro* show a developmental defect in connectivity and impaired dendritic growth observed at 7 and 21 days DIV. There is also a loss of cell viability, also suggestive of a neurodegenerative component to the FPM [70]. Interestingly, in the same neurons, the expression of the vesicular GABA and glutamate transporters VGAT and VGLUR1, respectively, is reduced at 21 DIV, but not at 7 DIV. These alterations are associated with a 4- to 8-fold increase in *Fmr1* mRNA and an approximately 50% decrease in FMRP.

Abnormal patterns of electrical activity are also seen *in vitro* in hippocampal neurons from CGG_dut_ KI mice, including enhanced clustered burst (CB) firing. Specifically, hippocampal neurons cultured from CGG_dut_ KI mice display CB electrical spiking activity and abnormal patterns of spontaneous synchronous Ca^2+^ oscillations under basal culture conditions [92]. The principal mechanisms contributing to these neuronal network defects in basal electrical activity appear to be associated with a gain of function in type I metabotropic glutamate receptors (mGluRs) and/or a loss of function in GABA_A_ receptor signaling. This conclusion is supported by data indicating that: the type I mGluR receptor agonist 3,5-Dihydroxyphenylglycine (DHPG), but neither NMDA nor AMPA receptor agonists, increased CB firing patterns in WT neurons with increased spike rate and mean burst duration similar to those observed in FPM hippocampal neurons; selective mGluR1/5 antagonists 7-(hydroxyimino)cyclopropa[b]chromen-1a-carboxylate ethyl ester (CPCCOEt) and 2-methyl-6-(phenylethynyl)pyridine hydrochloride (MPEP) abrogated abnormal electrical activity in FPM neurons; FPM astrocytes have impaired glutamate uptake [69,93]; WT cultures exposed to the astrocyte glutamate transport competitive antagonist DL-threo-β-benzyloxyaspartic acid produced electrical firing patterns indistinguishable from those of CGG_dut_ KI neurons; GABA_A_ receptor block with picrotoxin generated CB firing behavior observed in CGG_dut_ neurons; and the allosteric GABA_A_ receptor enhancer allopregnanolone essentially restored WT electrical spiking patterns.

These functional deficits are directly pertinent to the altered patterns of neuronal complexity reported earlier using the same *in vitro* CGG_dut_ KI model [70]. Neuronal network activity is essential for normal neuronal migration, dendritic growth and synaptic plasticity, processes mediated by spatially and temporally orchestrated intracellular Ca^2+^ signals. Therefore, the abnormal CB electrical activity and abnormal patterns of spontaneous Ca^2+^ oscillations observed in hippocampal neurons from CGG_dut_ KI mice are likely to contribute, at least in part, to impaired dendritic growth and synaptic architecture.

#### ***Hippocampal synaptic plasticity***

Deficits in processing spatial and temporal information have been reported in FPM carriers and in patients with FXTAS, suggesting hippocampal-associated pathology. In order to fully characterize the CGG KI mouse and to provide clues to which brain regions mediate these cognitive deficits (for example, hippocampus), *in vitro* studies of synaptic plasticity in acute hippocampal slices isolated from CGG_dut_ KI mice and WT mice have been carried out. Specifically long-term potentiation (LTP) of synaptic transmission and long-term synaptic depression (LTD) in CGG_dut_ and WT mice have been examined. The results demonstrated that the magnitude of LTP was significantly lower in CGG KI mice compared to WT mice, indicating impaired synaptic plasticity. Similarly, LTD, whether induced by low-frequency electrical stimulation (1 Hz) or bath application of the mGluR1/5 agonist DHPG, was also limited in CGG KI mice versus WT mice. These findings implicate loss of neuroplasticity in the hippocampus in the spatial and temporal cognitive deficits associated with CGG repeat expansions and the neurological pathology in FXTAS [94]. By contrast, enhanced LTD has been reported in the CGG_nih_ KI mouse model [95]. LTD at CA3-CA1 hippocampal synapses induced by bath application of the group I mGluR agonist DHPG was enhanced relative to that seen in WT littermates. *Fmr1* mRNA production was increased, FMRP translational efficiency in response to DHPG was impaired, and basal FMRP levels were moderately reduced. The authors noted that *Fmr1* KO mice completely lacking FMRP also showed enhanced LTD, suggesting that the enhanced LTD in the CGG_nih_ KI mouse may be due, at least in part, to lower levels of FMRP. The differing results for LTD between the CGG_dut_ and CGG_nih_ KI mouse models may therefore be the result of small versus moderate reductions in FMRP, respectively, indicating different cellular mechanisms for the differing results.

### Developmental aspects in FPM and FXTAS

FXTAS was originally described as a late-onset neurodegenerative disorder typically appearing in premutation carriers in the fifth or sixth decade of life. However, it is clear from both human [96,97] and mouse studies [29] that the consequences of the expanded CGG repeat can be seen in FPM carriers much earlier in development, indicating that the disease process likely begins much earlier in life, and possibly as early as during gestation [98]. Some children with the premutation have been reported to show cognitive deficits and behavioral problems, including symptoms of autism spectrum disorder and attention-deficit hyperactivity disorder [96,97]. Young (<12-week-old) CGG_dut_ and CGG_nih_ KI mice show impaired processing of spatial information [29] and abnormal locomotor activity and anxiety in the elevated Plus-maze [30].

The possibility that the premutation may affect early brain development is supported by findings in the CGG_dut_ KI mouse, where abnormal migration and differentiation of neuronal precursors during development of the embryonic cortical plate has been found [98]. In this study, precursor cells and embryonic neurons were labeled *in utero* on embryonic day 14 (E14) by intracerebral injections of a retrovirus encoding EGFP. The entire cell body, cytoplasm and processes of infected cells and their progeny were labeled with the EGFP reporter. The morphology of EGFP-labeled radial glial cells and immature neurons was not different between KI and WT neurons when examined on E17. However, there was evidence for altered differentiation of embryonic neural progenitor cells in the developing neocortex.

Radial glial cells in the ventricular zone express the transcription factor Pax6, divide at the ventricular surface and give rise to intermediate neuronal progenitor cells that express the transcription factor Tbr2 [99,100]. The CGG_dut_ KI mice had a greater number of Pax6^+^ cells in the ventricular zone and fewer Tbr2^+^ cells in the subventricular zone than WT mice, suggesting that delayed differentiation of the Pax6 cells in the CGG_dut_ KI mice may have produced a shift towards more Pax6^+^ and fewer Tbr2^+^ cells. Importantly, the shift in cell distribution could not be attributed to increased proliferation of Pax6^+^ cells, decreased proliferation of Tbr2^+^ or increased cell death among Tbr2^+^ cells. These data suggest that the *Fmr1* CGG repeat allele impacts the developing brain during gestation, much earlier than previously realized, and point to a neurodevelopmental component in FXTAS.

### Neurobehavioral correlates

Key features of patients with FXTAS are late-onset ataxia and memory impairments. Similar phenotypes have been found for CGG KI mice. Motor performance on the rotarod declines with age in CGG_dut_ KI mice [31]. In addition, sensory-motor coordination is impaired in adult CGG_dut_ KI animals when they are required to traverse a horizontal ladder (the ladder rung task). Both male and female CGG_dut_ KI mice showed impairments that were positively correlated with CGG repeat size [101]. Poor performance in the rotarod and ladder rung test may reflect the ataxia seen in FXTAS. Adult female CGG KI mice are also impaired in learning a skilled forelimb motor task, in which they are trained to reach through a narrow opening in a Plexiglas box to grasp and obtain a small food reward positioned just outside. Again, performance was worse with longer CGG repeat lengths [102]. Similar experiments have not yet been carried out in male mice. To date, studies in CGG KI mice have not reported intention tremors, a key neurological feature in FXTAS. The reason for this is unclear, but may be related to the quadrupedal organization of the rodent motor system.

Spatial learning and memory in the Morris water maze is impaired in CGG_dut_ KI mice at 52 weeks of age, but not at 20 weeks, indicating a progressive nature of the deficit [31]. Additional spatial deficits in CGG_dut_ KI mice are seen in the ‘metric’ spatial processing test, which involves processing precise angles and distances that separate objects in space, without regard to the identity of the objects [103]. In this test, mice are allowed to explore two identical objects separated in space by a fixed distance for 15 min (the study phase), showing very little further exploration at the end of this time. Mice are removed from the apparatus, the distance between the objects changed (for example, moved closer together), and the mice are allowed to re-explore the objects for 5 min (the test phase). During the test phase, WT mice showed increased object exploration, indicating that they detected a change in the distance between objects, whereas CGG_dut_ KI mice failed to re-explore the objects. Deficits in this task were seen as early as 12 weeks of age, when small but easily detectable intranuclear inclusions were present in neurons in the dentate gyrus of the hippocampus but not in the parietal cortex [29]. Lesion studies have implicated the dentate gyrus and CA3 hippocampus in processing of metric spatial information, a form of spatial pattern separation [104]. This suggests that histopathology (for example, presence of intranuclear inclusions, altered dendritic and spine morphology) in the dentate gyrus and CA3 subregion of the hippocampus in CGG KI mice may contribute to this spatial processing deficit. Although the role of intranuclear inclusions to pathology in FXTAS is unclear, the presence of intranuclear inclusions in different brain regions at different ages appears to follow a similar time course as the emergence of behavioral dysfunction in the CGG KI mouse, suggesting there might be a relationship between spatial deficits and inclusion formation [24,105,106].

Additional behavioral pathology found in the CGG_nih_ KI mouse model of the FPM and FXTAS includes mild hyperactivity, decreased anxiety in elevated plus maze, and impaired shock avoidance learning [30].

### Evidence for current disease models

#### ***RNA toxicity***

Studies in mouse models have been particularly useful in identifying molecular mechanisms in the FPM and FXTAS. An RNA ‘toxic gain of function’ mechanism has been proposed in which elevated *FMR1* mRNA transcripts bearing an expanded CGG repeat are cytotoxic. Toxicity appears to be the result of the expanded CGG repeat *per se*, and not of overexpression of *FMR1*. This is supported by the fact that ectopic expression of a CGG repeat expansion in the premutation range is sufficient to induce formation of intranuclear inclusions, reduce cell viability, trigger neuronal death (for example, Purkinje cell loss) and produce behavioral deficits [34,59,107], whereas overexpression of *Fmr1* mRNA without a CGG repeat expansion does not appear to be toxic [41]. Similar RNA toxicity has been suggested to underlie the pathology in several repeat diseases, including the myotonic muscular dystrophies. In this model, sequestration of important proteins through their interactions with expanded repeats prevents the proteins from carrying out their normal functions. As shown in Figure 2A, a similar protein sequestration mechanism has been proposed to underlie disease processes in the FPM and in FXTAS [2,36,82,108]. Based on studies in human and animal (for example, mouse, fly) tissues, a number of candidate RNA binding proteins have been identified, including DGCR8 and DROSHA [47], SAM68 [19], purα [109,110], hnRNPA2/B1 and CUGBP1 [37].

#### ***Sequestration of DROSHA/DGCR8 and miRNAs***

While the evidence is strong for the binding of proteins to CGG expansions and sequestration of proteins within ubiquitin-positive inclusions, the consequences of sequestration for cell function remain to be described. However, a recent study has linked sequestration of proteins associated with miRNA processing with the disease process in FXTAS [47]. Specifically, the double-stranded RNA-binding protein DGCR8 binds preferentially to CGG repeats of pathogenic length (that is, CGG repeat length >60). As depicted in Figure 2A, this leads to partial sequestration of DGCR8 and its binding partner DROSHA to expanded CGG repeats within CGG RNA aggregates. DGCR8 and DROSHA are important for processing pre-miRNAs into mature miRNAs by the DICER enzyme. Dgcr8 deficiency in heterozygous Dgcr8+/- mice results in reduced synaptic potentiation in layer five pyramidal neurons in the medial prefrontal cortex of mice [111]. Large deletions in the 22q11 locus, which include Dgcr8, result in altered dendritic spine morphology, reduced dendritic branching complexity and impaired working memory [112]. Similarly, loss of DICER in mice results in progressive neuronal degeneration [113], reduced dendritic branching and increased dendritic spine length [114], ataxia, and reduced brain size following deletion from striatal neurons [115]. These results suggested a model in which double-stranded CGG RNA forms hairpins [91] that mimic the RNA structure of pre-miRNAs recognized by DGCR8 [47]. DGCR8 and its partner DROSHA bind to the expanded CGG repeat element and are therefore sequestered, reducing the production of mature miRNAs causing neuronal dysfunction and death [47]. This possibility is supported by the observation that the expression of mature miRNAs was decreased in postmortem brain samples from patients with FXTAS. In addition, *in vitro* overexpression of DGCR8 restored normal dendritic growth and branching, and alleviated cell death of cultured neurons expressing a toxic 60 CGG repeat [47].

#### ***Repeat associated non-AUG translation***

An additional mechanism of toxicity is shown in Figure 2B. In this model, toxicity is triggered by CGG RAN translation [20]. This is based on evidence that trinucleotide repeats can be translated into protein even if they do not reside in an AUG-initiated open reading frame [116], and such translation can occur in all three possible open reading frames of a transcript generating multiple potentially toxic products from a single repeat [117]. In the case of FXTAS, it has been proposed that RAN translation initiated in the 5′UTR of *FMR1* mRNA results in the production of a cytotoxic polyglycine-containing protein named FMRpolyG [20]. This is supported by results from human FXTAS and animal model studies. Specifically, the presence of the FMRpolyG was confirmed by western blot in cerebellar lysates of postmortem FXTAS brains. FMRpolyG staining was specific for FXTAS, and was not found in control brains, or in brain sections from patients with spinocerebellar ataxia type 3 or Alzheimer’s disease. Interestingly, there were clear differences between the CGG_dut_ KI and CGG_nih_ KI mouse models, with co-localization of FMRpolyG and ubiquitin-positive intranuclear inclusions in the cortex and hypothalamus of the CGG_dut_ KI mouse, but not in the CGG_nih_ KI mouse. These data suggest that some of the differing pathology between the two mouse models could be explained by differences in the ability to generate the toxic polyglycine peptide. The mechanisms underlying RAN translation are as yet unknown, but the presence of the polyglycine peptide (that is, FMRpolyG) in FXTAS and the CGG KI mouse models led to the proposal by Todd *et al*. that a scanning 43S ribosomal pre-initiation complex stalls at the CGG repeat, resulting in use of an alternative non-AUG start site for translation in the +1 reading frame (that is, GGC, polyglycine) and the production of the FMRpolyG protein. The data did not show translation product from the +0 (that is, CGG, polyarginine) reading frame, but some, albeit less efficient, translation in the +2 (that is, GCG, polyalanine) reading frame was observed [20].

## Conclusions

Although uniquely human components of disease cannot be fully captured in other species, mouse models of FXTAS have provided useful research tools to test hypotheses about the causes of the disorder, and to discover effective treatments. Development of CGG KI mice has provided insight into the natural history of the disorder, the molecular correlates, hallmark pathology in the brain and other organ systems, as well as an understanding of the neurobehavioral effects of expression of CGG repeat expansions. These KI mice now allow for the evaluation of novel therapeutic strategies, whether pharmacological or gene-targeted, to halt or reverse disease processes and to improve neurological outcome. Ongoing development of new mouse lines, including conditional and inducible mice, should further increase the value of animal models to understand the pathology of repeat disorders such as FXTAS. There are many open questions to be answered that will continue to rely on mouse models, including why mRNA levels are elevated, the importance of reduced FMRP in pathology, whether intranuclear inclusions are toxic or simply mark the progress of disease, and how protein sequestration and RAN translation contribute to the disease process in FPM and FXTAS.

## Abbreviations

AMPA: α-amino-3-hydroxy-5-methyl-4-isoxazole propionic acid; ATPB: β-subunit of ATP synthase; CB: clustered burst; DHPG: 3,5-dihydroxyphenylglycine; DIV: days *in vitro*; EGFP: enhanced green fluorescent protein; FAD: flavin adenine dinucleotide; FMRP: fragile X mental retardation protein; FPM: fragile X premutation; FXS: fragile X syndrome; FXTAS: fragile X-associated tremor/ataxia syndrome; KI: knock-in; KO: knock-out; LTD: long-term synaptic depression; mGluR: metabotropic glutamate receptors; miRNA: microRNA; MnSOD: manganese superoxide dismutase; NMDA: N-methyl-d-aspartate; NAD: nicotinamide adenine dinucleotide; UTR: untranslated region; WT: wild-type; YAC: yeast artificial chromosome.

## Competing interests

DLN patented a method for detecting *FMR1* mutation: US PTO 6,107,015 diagnosis of the fragile x syndrome. All other authors declare that they have no competing interests.

## Authors’ contributions

All of the authors contributed to the conceptualization and the writing of this review. All authors read and approved the final manuscript.
